# EcoDaLo: Federating Advertisement Targeting with Linked Data

**DOI:** 10.1007/978-3-030-59833-4_6

**Published:** 2020-10-27

**Authors:** Sven Lieber, Ben De Meester, Ruben Verborgh, Anastasia Dimou

**Affiliations:** 8 Linköping University, Linköping, Sweden; 9 University of Amsterdam, Amsterdam, Noord-Holland The Netherlands; 10 Department of Computer Science, Vrije Universiteit Amsterdam, Amsterdam, Noord-Holland The Netherlands; 11 St. Pölten University of Applied Sciences, St. Pölten, Austria; 12 FIZ Karlsruhe – Leibniz Institute for, Karlsruhe, Germany; 13 Karlsruhe Institute of Technology, Karlsruhe, Germany; 14 UAS St. Pölten, St. Pölten, Niederösterreich Austria; 15 Vienna University of Economics and Business, Vienna, Wien Austria; 16 VU Amsterdam, Amsterdam, The Netherlands; 17 ADAPT Centre, Trinity College Dublin, Dublin, Ireland; Department of Electronics and Information Systems, Ghent University – imec – IDLab, Technologiepark-Zwijnaarde 122, 9052 Ghent, Belgium

**Keywords:** Advertisement, Federation, Linked Data

## Abstract

A key source of revenue for the media and entertainment domain is *ad targeting*: serving advertisements to a select set of visitors based on various captured visitor traits. Compared to global media companies such as Google and Facebook that aggregate data from various sources (and the privacy concerns these aggregations bring), local companies only capture a small number of (high-quality) traits and retrieve an unbalanced small amount of revenue. To increase these local publishers’ competitive advantage, they need to join forces, whilst taking the visitors’ privacy concerns into account. The EcoDaLo consortium, located in Belgium and consisting of Adlogix, Pebble Media, and Roularta Media Group as founding partners, aims to combine local publishers’ data without requiring these partners to share this data across the consortium. Usage of Semantic Web technologies enables a decentralized approach where federated querying allows local companies to combine their captured visitor traits, and better target visitors, without aggregating all data. To increase potential uptake, technical complexity to join this consortium is kept minimal, and established technology is used where possible. This solution was showcased in Belgium which provided the participating partners valuable insights and suggests future research challenges. Perspectives are to enlarge the consortium and provide measurable impact in ad targeting to local publishers.

## Introduction

*Digital advertising* is the act of serving advertisements (“ads”) in different formats to *visitors* who consume online content on *publishers*’ websites. It is a key source of revenue in media and entertainment domain: *advertisers* that set up an *ad campaign* receive revenue from the company ordering the campaign, and publishers receive money from advertisers to display ads. When setting up an ad campaign, advertisers specify which and how many ads are served (from one or more companies) as well as its format.

In *ad targeting*, an advertiser also defines a pre-selected set of visitors based on various *traits*, e.g. geography, demographics, psychographics, browsing behavior, or past purchases. Ad targeting increases the probability of a visitor reacting positively compared to serving the same ad to every visitor [30], and, thus, results in higher return on investment for both publishers and advertisers.

The *profile*, the trait set of a visitor, needs to be *captured*, using *observations* via various complementary channels. For example, when Alice visits the sports page of a publisher’s website more than eight times per month, that publisher – or a third-party tracker – adds the trait “liking sports” to Alice’s profile (*web browsing behavior* observation). When Alice registers herself on that website and enters her birth date, her age range trait (e.g., 40–55) is also added to her profile (*demographics* observation). Alice can be targeted by the profile *“People over 35 years old liking sports”*, as her profile matches, as long as sufficient consent was provided upfront by Alice. When more traits of Alice are captured, she can be targeted by more (and more specific) ads.

However, profile data, and the revenue they entail, are unevenly distributed [3]: it was predicted that, in the first quarter of 2016, 85% of online advertising spendings would go to either Google or Facebook [14]. Such global publishers are media conglomerates and track visitors far beyond their own media properties. It is estimated that at least 68% of the most popular websites are tracked by Google [10]. These companies aggregate and centralize a large amount of data, and enable advertisers to create rich profiles. In contrast, local publishers hold only a fraction of visitor traits, as found on their own websites. Those traits are typically of higher quality compared to global companies, as local publishers have a closer relationship with their visitors. However, local publishers typically miss the opportunity to target visitors matching a requested profile, due to lack of scale, and, hence, miss out on revenue.

Combining multiple local publishers’ data can improve the profiling information and make their generated profiles – due to higher quality – competitive to global publishers. However, aggregating and centralizing all data understandably comes with limitations. Recent large-scale data scandals made the general public increasingly aware of the importance of privacy and control over personal data. The introduction of the General Data Protection Regulation (GDPR) in the European Union [9] enforces explicit, freely-given consent for sharing personal data. More, sharing all data across publishers would not be well received by the publishers, as this would result in loss of competitive advantage. The data should thus remain exclusive to each publisher.

Using *federated querying* the data remains spread among – and under control of – publishers. However, it allows discovering visitors that adhere to a certain targeted profile, combining the relevant data from multiple publishers via federated querying. Linked Data [1] acts as an enabling technology: (i) the interoperable layer allows uniform and unambiguous trait descriptions across publishers and (ii) richer profiles are created via federated querying, while the data does not need to be shared across publishers. The usage of semantic technologies, thus, allows local publishers to join forces, leveling the playing field with global companies. Local publishers and advertisers do not need to fully share their data, whilst improving ad targeting.

A solution based on federated querying is devised mapping publisher’s custom trait definitions to a common SKOS vocabulary [20], generating RDF datasets using RML [7] and queried using Comunica [26]. This solution is applied to and deployed in the media landscape of Flanders, Belgium, as it is explained at https://vimeo.com/374617281. A consortium was formed, dubbed *EcoDaLo*, consisting of complementary partners to deploy this interoperable layer: Adlogix, Pebble Media, and Roularta Media Group.

We present the role semantic technologies play in EcoDaLo, allowing federated advertisement targeting in Belgium. After introducing the use case (Sect. 2), we present our application (Sect. 3). Our approach was showcased by multiple companies in Belgium (Sect. 4), allowing federated integration of traits to improve targeting across local publishers. We functionally evaluate our solution (Sect. 5), present related work (Sect. 6), and conclude by discussing privacy and ethical considerations as well as key features of our solution (Sect. 7).

## EcoDaLo

The EcoDaLo consortium is one of the first collaborations where publishers remain in exclusive control of data they collected, and a decentralized deployment is attained. Three complementary funding consortium partners participate in EcoDaLo. **AdLogix** is a development company experienced in digital advertising, which developed multiple advertising products on the international market^1^. It is responsible for providing technical support to build a production-ready system that can be used by both advertisers and publishers. **Pebble Media** is a digital sales house, representing the role of advertiser, with many partnerships in the local market^2^. **Roularta Media Group** is a multimedia group, representing the role of publisher, and market leader in the field of radio and television, magazines, and local media in Flanders^3^. As domain experts, Pebble Media and Roularta Media Group are responsible for providing technical requirements, aligned with the current advertising industry landscape. As all bases are covered by the different consortium partners, the devised solution remains in line with industrial perspectives, and chances of successful impact increases.

**Fig. 1.**
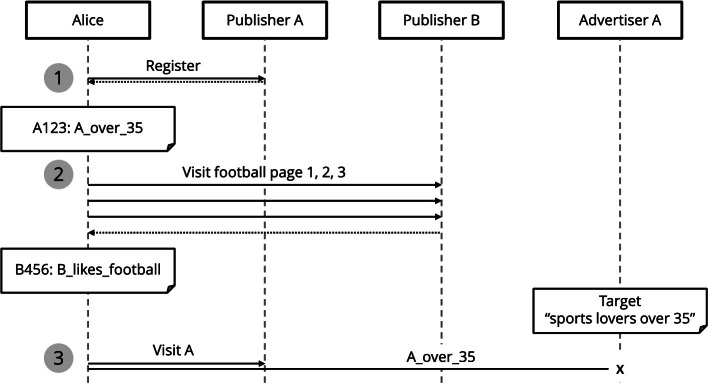
Publisher A knows Alice is over 35 years old, and Publisher B that she likes football. However, Alice cannot be targeted, as her captured traits from different publishers cannot be combined.

**Fig. 2.**
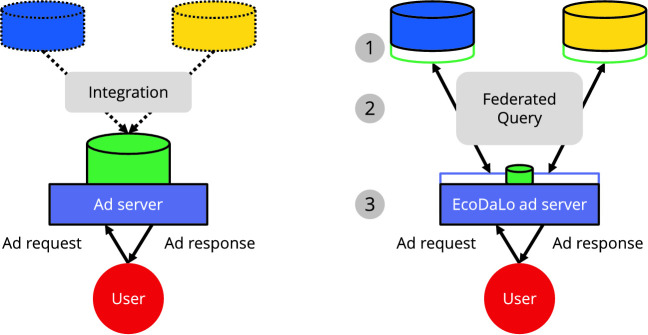
Current practice (left) vs. EcoDaLo (right). Typically, all captured data is integrated in a common ad server (center-left), and publishers give up control of their data (dotted DBs, top-left). In EcoDaLo, a common ad server only captures visitor identification data (small DB, center-right). Publishers require an additional layer enabling federated querying (extra DB outlines, top-right).

**Fig. 3.**
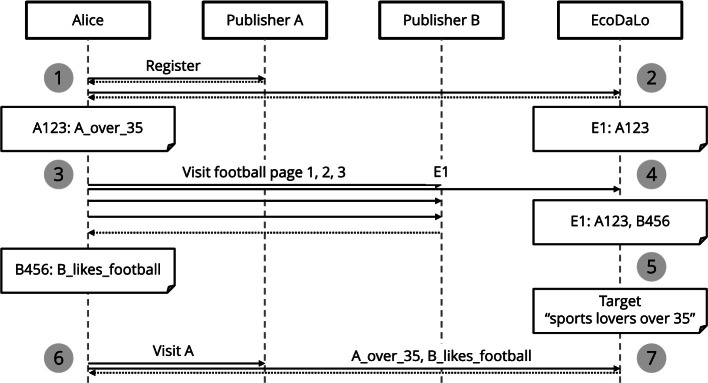
Alice is targeted by combining multiple traits, from different publishers.

*Motivating Example.* Alice visits the websites of publishers A and B (Fig. 1). The publishers have different ways of identifying Alice’s traits. Publisher A knows her age range because Alice registered her birth date: Alice is identified with id *A123* and she gets assigned trait *A_over_35* (Fig. 1, *1*). Publisher B – specialized in football content – deduces that Alice is football lover, because she visits any of the publisher’s web pages more than once a week: Alice is identified with id *B456*, she gets assigned trait *B_likes_football* (Fig. 1, *2*). None of the publishers can provide enough traits to match Alice to *“sports lovers over 35”* (Fig. 1, *3*). And even if publishers could combine their user traits, it is not clear whether a football lover qualifies as a sports lover or not. EcoDaLo aims to enable this potential, semantically – i.e., meaningfully – combining the captured data, and serve Alice relevant advertisements, targeted at the requested profile.

## Federating Advertisement Targeting with Linked Data

EcoDaLo aims to improve ad targeting by combining visitor data *across publishers*. This allows leveling the playing field between local and global publishers: local publishers can target more visitors, and their captured visitor traits are of higher quality compared to those of global publishers. Typically, integrating all publishers’ data results in an additional ad server having access to a large amount of data. This provides a global fine-grained view of every individual visitor, and allows detailed analysis over all data. However, it also requires publishers to give up control over the data they captured (Fig. 2, left).

The addressed challenges include cross-publisher targeting without sharing all data and providing an extensible and scalable framework to various new partners. We chose to keep the data spread across publishers, and let a separate neutral party do federated querying on the level of captured visitor traits, using unambiguous semantic descriptions, instead of integrating all publisher’s individual observations. For example, not every observation that Alice visits a football page is shared across publishers, only Publisher B’s (aggregated) captured trait that Alice likes football is taken into account during federated querying. Also the aggregated captured trait is not shared with other publishers, it is only taken into account by the federated query layer.

The combination of federated querying, and only considering the captured visitor traits instead of all data, alleviates *privacy concerns*, improves *scaling behavior*, and *exploits existing infrastructure*. The disadvantage is that ad targeting by combining visitor traits is not as fine-grained as integrating all data. For example, it is not possible to target visitors that “visited at least three sports pages across all publishers in the last 10 days”, as such information is not shared.

Visitors’ *privacy* is protected to a certain extent: no fine-grained information is shared across consortium partners. Visitors are, to this point still, identifiable across publishers, but the captured traits (and links from these traits to unique visitors) remain under (exclusive) control of the publishers. The business rules of how those traits are captured remain exclusive to the individual publishers.

The solution *scales* as less data needs to be federated: a captured trait can be an aggregation from a large number of historical observations. Considering only the aggregations can reduce the amount of data by multiple orders of magnitude.

Publishers’ *existing trait capture infrastructure* is reused, compared to installing a large new trait capture infrastructure. The existing infrastructure – optimized to aggregate large amounts of (historical) data to capture traits – remains unaltered: its output, i.e., the discovered visitor traits, serves as a data source for the federated querying. This reduces development effort for the consortium partners, and increases the chances of adoption by more publishers.

In this section, we provide a high-level overview (Sect. 3.1) and an example (Sect. 3.2), after which we discuss our design considerations (Sect. 3.3).

### High-Level Overview: Federated Querying with Common Identifier

Our solution consists of three main components (Fig. 2, right):

(i) The *EcoDaLo ad server* – auxiliary to the pre-existing ad servers used by each respective publisher – targets and serves ads to visitors across publishers (Fig. 2,  ). This ad server only provides the common identifier; the visitor traits remain under the individual publishers’ control.
(ii) Each publisher provides a *semantic layer*, exposing the captured visitor traits mapped to an interoperable unambiguous trait model (Fig. 2,  ).
(iii) A *federated querying* intermediate layer connects the additional ad server with the individual publishers (Fig. 2,  ). Due to the explicit semantics, we provide an interoperable layer, extensible to new partners.

### Example of Federated Querying with Common Identifier

Using our solution, Alice can be targeted by combining multiple traits from different publishers (Fig. 3). Alice visits a website of Publisher A as a registered visitor (Fig. 3,  ). She is identified as new visitor within EcoDaLo (EcoDaLo id *E1*,  ). Alice then browses some football pages of Publisher B as an unregistered user  . She is recognized as existing visitor within EcoDaLo  .

When a new campaign is launched, the trait combinations are queried, federated over the different publishers  ). The mapping to a common trait model is used to query the individual publisher’s captured traits, e.g., *over_35* is found mapped from *A_over_35*, and *sports_lover* mapped from *B_likes_football*.

When Alice then visits a consortium publisher, such as Publisher A, her set of captured visitor traits is sent to the EcoDaLo ad server  . Alice’s trait set matches with the mapped target set, Alice is targeted by the campaign, and a relevant ad is served  . Her EcoDaLo id *E1* makes sure the number of times Alice gets served a specific ad is monitored correctly, even when she visits Publisher B. During *ad targeting*, Alice is not uniquely identified, i.e., her EcoDaLo id is not used during federated querying, only during *ad serving*. Thus, the explicit link between Alice and her captured traits is never stored in the EcoDaLo ad server.

### Design Considerations

Each publisher has its own trait definitions. This influences ad campaign definitions and visitor targeting. Before defining an ad campaign, a common, unambiguous trait model is needed for the traits targeted by advertisers, those captured by publishers, and the relationships between them. For example, Publisher A captures three age ranges (“<18”, “18–35”, “>35”), and Publisher B captures five age ranges (“<18”, “18–25”, “25–35”, “35–65”, “>65”): the targeted trait “over 35” is mapped differently for Publisher A and Publisher B.

A semantic model further allows description of trait relations, e.g., the relationship between Publisher B’s captured “likes football” and the more general “sports lover” can be specified. Instead of requiring all consortium partners to alter their system and impose usage of a common trait model, publishers map their existing captured traits to a common model. This *increases flexibility*: a single captured trait can be mapped to multiple common traits, and a combination of captured traits can be mapped to a single common trait. This increases the chances of adoption as changes in the publishers’ pre-existing infrastructure and required effort are minimized.

A visitor can thus be targeted by combining the captured traits across publishers, and served a relevant ad. However, to monitor how many ads are served to how many distinct visitors, a shared identification mechanism is still needed. Multiple options were considered to identify visitor across publishers, among others, machine learning and browser fingerprinting:

*Machine learning* techniques could help identify individual visitors based on their combination of traits. However, more detailed data is not available – given that no fine-grained observations are shared – and this would require to create a training set of visitors and addressing the related emerging privacy concerns.

*Browser fingerprints* [18] provide a quasi-unique identification mechanism by combining visitors’ browser and hardware traits, e.g., installed plugins, screen resolution, etc. The identification is not 100% accurate, and identification is limited to visitors using a single browser and device.

However, these options were dismissed due to the inability to provide 100% accurate results. Given the domain, where inaccuracies are already manifold (e.g., visitors using multiple devices, sharing the same accounts, etc.), the consortium decided not to add more inaccuracies. Instead, we use the EcoDaLo ad server as identifying service, which provides and explicitly links common (EcoDaLo) ids to the visitor ids of each consortium partner.

The ad server only stores its own generated ids, mapped to the ids of the individual publishers. For example, when Alice first visits Publisher A, she is not yet identified within EcoDaLo, the ad server creates a new common EcoDaLo id *E1*, and connects this id with *A123*, Alice’s id of Publisher A (Fig. 3,  ). When Alice later visits Publisher B, given her previously assigned EcoDaLo id *E1*, the ad server is updated and Publisher B’s id *B456* is added (Fig. 3,  ).

## Deployment

EcoDaLo’s technical considerations include setting up the EcoDaLo ad server (Sect. 4.1), using a common trait model (Sect. 4.2), mapping each publisher’s traits to that common trait model (Sect. 4.3), federating the traits (Sect. 4.4), and exposing the results to the EcoDaLo ad server (Sect. 4.5). The (development) effort for partners to integrate with the EcoDaLo set-up is kept low to increase potential uptake and growth of the consortium.

### EcoDaLo Ad Server

The EcoDaLo ad server: (i) provides common visitor ids across consortium partners, (ii) serves ads of campaigns set up within the EcoDaLo consortium, and (iii) monitors the number of ads served to distinct visitors. As such, established pre-existing ad server software can be used to fulfill multiple requirements. We employ an ad server that provides identifiers for every visitor of any website within the consortium. Each publisher needs to modify its websites, allowing access to the EcoDaLo ad server to add these identifiers.

The expected effort is reasonable, as the publishers would need to support ads served due to campaigns set up in EcoDaLo in any case. Publishers that advertise are already required to gather GDPR-compliant visitor’s consent for ad targeting involving third-parties, i.e. informing the user who will have access to which information for which purpose. Thus, no additional effort regarding the consent gathering setup is needed compared to existing solutions.

### Common Trait Model

We use an interoperable, semantic model to describe the common traits, as it enables meaningful federation across publishers. We provide a Simple Knowledge Organization System (SKOS) taxonomy [20] based on the IAB Technology Laboratory’s Audience Taxonomy 1.0^4^ as common trait model. The IAB Technology Laboratory (IAB Tech Lab) is an international nonprofit consortium that helps companies implement global advertising industry technical standards and solutions. We only considered IABs audience taxonomy as a possible common trait model, other trait models can be used or created instead. This taxonomy is available at http://semweb.mmlab.be/ns/iab/at_1-0, mapped from the originally published taxonomy to SKOS using YARRRML [6, 15], and processed as RML rules [7, 8]. The mapping rules are available at http://semweb.mmlab.be/ns/iab/mapping/iab_audience.mapping.yaml and http://semweb.mmlab.be/ns/iab/mapping/iab_audience.mapping.rml.ttl.

The modeling effort is limited compared to the typical approach where all publishers’ data is integrated: only the traits need to be modeled, as opposed to all types of publisher observations and descriptions of how observations lead to a captured trait. For example, we do not need to model that the set of observations “visiting at least three football pages the last 10 days” is used to capture the “football lover”-trait. The use of a declarative mapping language allows for possibly fine-grained mappings including the use of functions but can also be created manually in a hard-coded fashion. In any case, we provide a transparent and maintainable process, adaptable for change, as the Audience Taxonomy is currently released for public comment.

### Mapping to the Common Trait Model

Each publisher is required to provide a mapping of the captured internal traits to the common ones. This mapping can be many-to-many, across multiple levels. For example, “football lover” is mapped to “Sports—American Football” and the more general “Sports”, and “tennis lover” is – next to “Sports—Tennis” – also mapped to “Sports”. More granular mappings can be taken into account, e.g., distinguishing the levels of interest of a “football lover”.

The Resource Description Framework (RDF) [5] is useful to describe the mapping, as it natively allows to unambiguously link concepts in complex relationships. For usability reasons, consortium partners – which are non-Semantic Web experts – do not need to manually write RDF triples. Instead, they provide a mapping of their custom captured traits to the common trait model, by means of a CSV file with three columns: the publisher’s captured internal trait id and label, and the common trait_id from the IAB Audience Taxonomy.

This CSV file is then used to generate the RDF dataset mapping each publishers’ internal traits to the common trait model. The generation description is written in YARRRML [6, 15], a representation of RML [7, 8] (Listing 1): the generation process remains maintainable, whilst consortium partners are not bothered with the details of how RDF triples are generated. Every time the mapping changes, i.e., when a publisher captures new visitor traits, the RDF dataset is regenerated and republished. 
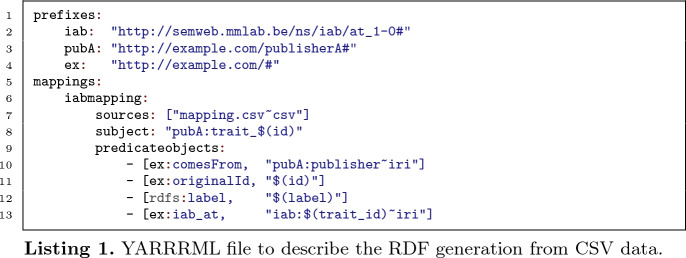


We provide a transparent and maintainable generation process, adaptable for change, by using RML. The generation description remains user-friendly relying on a CSV configuration document: CSV is easily handled using standard office suites, and a common export format for many software packages.

### Federated Querying Layer

Federated querying of cross-publisher traits is enabled using the generated interoperable RDF datasets of each publisher. Each publisher’s mapping dataset is generated in HDT format [11, 19], and published as a Triple Pattern Fragments (TPF) endpoint [29]. The federated query engine Comunica [26] queries over the TPF endpoints of each publisher, and over the published SKOS Audience Taxonomy. An example of a federated query for all captured traits is shown in Listing 2. The traits are found across all publishers (line 11), and returned with their preferred label from the published SKOS Audience Taxonomy (line 15). 
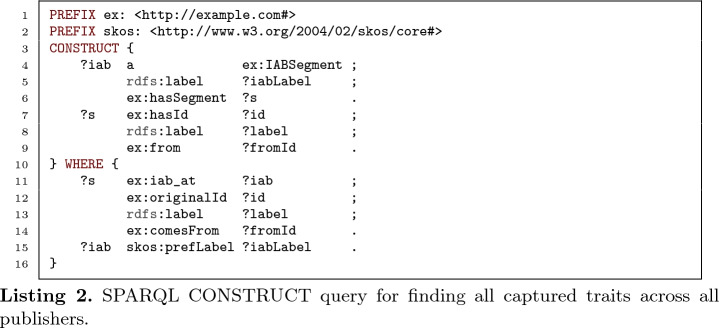


Publishing results using TPF endpoints in lower server-side CPU usage – thus requiring minimal investment of the consortium partners – and – in combination with Comunica – delivers state-of-the-art federated querying performance [29].

### Developer-Friendly API

A JSON(-LD) [25] API is provided that exposes the results of federated SPARQL queries, easing integration with the EcoDaLo ad server. The JSON-LD context hides the individual URI prefixes. This API is consumed (daily) by the EcoDaLo ad server, to have an updated view of the consortium partners’ captured traits.

In an initial stage, the complexities of using RDF are hidden from the partners, which lowers the threshold for new partners to join the consortium: no prior Semantic Web knowledge is needed.

## Validation

The consortium collected focus groups to make sure the devised solution is in line with the industry’s common practices. All decisions were communicated in face-to-face meetings, and feedback was gathered using the think-aloud method [24]. We discuss a launched campaign that evaluate the added benefit of EcoDaLo and compare EcoDaLo to other approaches based on six identified features.

### Launched Campaign

Our devised solution reached Technology Readiness Level (TRL) 5: we implemented and validated it in a relevant environment within a launched advertisement campaign in the end of August 2019 in Flanders, Belgium.

The Belgian university Vrije Universiteit Brussel (VUB)^5^ acts as client in the launched campaign and wants to target (potential) students to maximize the registrations for the open VUB day on September 7th 2019^6^. The campaign targets (combinations of) both overlapping and complementary captured visitor traits of both Roularta Media Group and Pebble Media in different advertising formats, such as “half page” or “mobile leaderboard”. Additionally we measured the traffic to the website of the open day at VUB using a tracking pixel.

Our devised solution has been presented to the industrial partners and served as technological base for the described campaign. Around 1.84 million impressions were delivered by Pebble Media and 1.03 million via Roularta Media Group; VUB reported that compared to last year 300 extra people were registered for the open day. Additionally industry partners using our solution reported insights in different renumeration models, i.e. how to split revenue based on provided knowledge about visitor traits and advertising format of the impression.

### Functional Comparison

To evaluate the added benefit of EcoDaLo, we perform a functional evaluation of six features, comparing EcoDaLo (*trait federation*) to the status quo of a *local publisher*, a *global publisher*, and an *integration* approach (Table 1).

**Table 1.** Summary of functional comparison of advertisement approaches with respect to a set of identified features.

	Feature	Local publisher	Global publisher	(Data) integration	(Trait) federation
1	Trait quality	$++$	−	$++$	$++$
2	Scale	−	$++$	$+$	$+$
3	Exclusive (privacy)	++	−	−	$+$
4	Ease of set-up	++	++	−	+
5	Interoperability	$-{}-$	$-{}-$	−	$++$
6	Maintainability	−	−	−	+

- **Trait quality.** The *trait quality* of a local publisher is – due to the locality – higher compared to those of a global publisher. This high quality is retained when integrating the captured data or federating the traits.
- **Scale.** The number of visitors that can be targeted, and the number of different traits that can be captured, i.e., the *scale*, increases during integration and federation, however, it does not necessarily reach the same numbers as for global publishers.
- **Exclusive.** Captured data is shared with a global publisher or during integration: it is no longer *exclusive* to a single local publisher. During federation, only common traits are shared with the EcoDaLo ad server, the visitor’s privacy with respect to all collected data is considered.
- **Ease of set-up.** Federation requires only the mappings of aggregated traits compared to integration where all observation types must be mapped to a common model; which still requires effort but less.
- **Interoperability.** Integration slightly improves *interoperability* by using common definitions, as compared to the closed environment of local and global publishers. However, the Linked Data principles renders the federation approach entirely interoperable and machine-understandable.
- **Maintainability.** Attention was put into improving the *maintainability* of the federation approach, specifically, into maintainability of the common trait model generation and the trait mapping description.

## Related Work

We describe related work regarding privacy, semantic web and advertisement.

Online behavioral advertisement (OBA) is controversial: on the one hand, it creates more relevant and efficient ads, on the other hand raises privacy concerns as it is based on personal data. For a complete overview of the topic we refer the reader to the literature review of Boerman et al. [2].

The W3C Data Privacy Vocabularies and Controls community group developed a vocabulary to annotate and categorize instances of legally compliant personal data handling [23]. This is complementary to our solution as their vocabulary describes consent and data processing purposes in EcoDaLo.

The SPECIAL project proposed a privacy-aware big data architecture focused on consent management and compliance verification [17]. It was developed in parallel with EcoDaLo. SPECIAL’s sticky privacy policies, data use constraints attached to data, could be realized within EcoDaLo by also mapping consent-related information from publishers to a common data model, similar to visitor trait data, providing the added feature of ex-ante compliance checking.

Publishers join forces by introducing an integration component that allows aggregating all involved publishers’ captured data [21]. This requires considerable development effort, tailored to existing publishers’ data stores and detailed privacy-compliance considerations. As such, a federated approach for querying captured visitor traits is, to the best of our knowledge, novel for ad targeting.

Usage of Semantic Web technologies to enable trait federation in the media and entertainment domain was not yet investigated. Existing related work instead focuses on automatically generating meaningful targeting profiles, by (i) *classifying* content and ads to form one knowledge graph, and (ii) using that knowledge graph to improve ad *recommendation* algorithms:

The semantic *classification* is either created manually [28], or content and ads are classified automatically to a common predefined knowledge graph [4]. Choosing between manual or automatic classification typically introduces a trade-off between quality and scalability. When improving the quality of the automatic classification, existing Linked Open Data graphs are used to complete the knowledge graph [13], and the explicit semantics are exploited to provide detailed tagging of content and ads [12]. During *recommendation*, typically, graph distance metrics are used as a measure of relatedness [31], an approach applied successfully in the academic publishing domain [27].

For EcoDaLo, ad targeting profiles are created manually by the advertiser. Related work is thus complementary, enabling improvements as future work: recommendation methods can be used to suggest inclusion or exclusion of certain traits when specifying an ad campaign.

## Conclusion

Advertising is a monetary stimulus for individuals to share their data with publishers and advertisers, in exchange for content. Although not the only option, it is very common in the media and entertainment domain. Lately, awareness rises concerning the trade-off between respecting an individual’s privacy and increasing advertising revenue. In EcoDaLo, we introduce an interoperable semantic layer among local publishers allowing to exploit high-quality visitor traits using federated querying, without sharing data among consortium partners.

We conclude by discussing privacy and ethical considerations, key features of our approach as well as outlining future work.

### Privacy and Ethical Considerations

The misuse of personal data, especially for discrimination, is unethical and illegal; *transparency* and *ethical guidelines* may address this issue.

Intransparency regarding the use of data collected via online behavioral advertising may be harmful and unethical if consumers are unaware [2]. The GDPR addresses transparency with respect to user-awareness about *which personal data*^7^ is shared with *whom* and for which *purpose* by listing obligations regarding valid consent obtainment. Recent court rulings applied these regulations on concrete cases [16] emphasizing on explicit opt-in to give consent.

For EcoDaLo, users need to be aware which personal data of which EcoDaLo publisher is used for the purpose of online advertisement, including awareness regarding participating publishers. Users then have to explicitly give consent for this purpose, i.e. they explicitly have to opt-in. EcoDaLo assumes that publishers and advertisers act with good faith following relevant ethical guidelines [22] which goes beyond the presented technical solution.

### Key Features of Our Approach

Hiding the complexities of using semantic technologies increases the potential uptake by new consortium partners. Consortium partners are not confronted with RDF triples or ontologies but, instead, rely on developer-friendly formats such as CSV and JSON. The federated querying layer and interoperable machine-understandable model are made transparent, lowering effort for consortium partners and increasing chances of enlarging the consortium. Although explicit semantics are currently hidden for consortium partners, (future) advantages are gained, compared to using an ad-hoc integration layer. Unambiguous machine-understandable trait definitions increase interoperability, and make it easier for new members to join the consortium. Reasoning can be applied to automatically enrich knowledge graph: implicit links between common traits can be discovered.

### Future Work

For future work, we investigate in a complementary validation component of the federated querying which i.a. can filter results which are too narrow and could harm privacy. Also, we look into the influence of using fine-grained traits and applying more advanced queries, a.o., taking into account a captured trait’s confidence level. For example, when the trait “likes sports” is captured with a low confidence level by multiple publishers, this can be combined as a single “likes sports” trait with higher confidence.

## Footnotes

1 http://www.adlogix.eu/.

2 http://www.pebblemedia.be/.

3 https://www.roularta.be/en.

4 https://bit.ly/3fqVVko, hosted locally at https://bit.ly/2zfhkwe.

5 https://www.vub.be/en/home.

6 https://www.vub.be/events/2019/infodag-7-september-2019.

7 GDPRs definition of personal data also partially overlaps with protected characteristics related to discrimination and thus ethics: https://www.equalityhumanrights.com/en/equality-act/protected-characteristics.

## References

[1] Bizer C, Heath T, Berners-Lee T (2011). Linked Data - the story so far. Int. J. Semant. Web Inf. Syst..

[2] Boerman SC, Kruikemeier S, Borgesius FZ (2017). Online behavioral advertising: a literature review and research agenda. J. Advert..

[3] Bond, S.: Google and Facebook build digital ad duopoly. Financial Times (2017)

[4] Broder, A., Fontoura, M., Josifovski, V., Riedel, L.: A semantic approach to contextual advertising. In: Proceedings of the 30th Annual International ACM SIGIR Conference on Research and Development in Information Retrieval (2007)

[5] Cyganiak, R., Wood, D., Lanthaler, M.: RDF 1.1 concepts and abstract syntax. Recommendation, World Wide Web Consortium (W3C) (2014). http://www.w3.org/TR/rdf11-concepts/

[6] De Meester, B., Heyvaert, P., Dimou, A.: YARRRML. Unofficial draft, imec – Ghent University – IDLab (2019). https://w3id.org/yarrrml/spec

[7] Dimou, A., Vander Sande, M.: RDF Mapping Language (RML). Unofficial draft, Ghent University - iMinds - Multimedia Lab (2014). http://rml.io/spec.html

[8] Dimou, A., Vander Sande, M., Colpaert, P., Verborgh, R., Mannens, E., Van de Walle, R.: RML: a generic language for integrated RDF mappings of heterogeneous data. In: Proceedings of the 7th Workshop on Linked Data on the Web (2014)

[9] Directorate-General for Justice and Consumers: The GDPR: New opportunities, new obligations: what every business needs to know about the EU’s General Data Protection Regulation. Eu Publications, European Commission (2018)

[10] Englehardt, S., Narayanan, A.: Online tracking: a 1-million-site measurement and analysis. In: Proceedings of the 2016 ACM SIGSAC Conference on Computer and Communications Security, pp. 1388–1401 (2016)

[11] Fernández JD, Martínez-Prieto MA, Gutiérrez C, Polleres A, Arias M (2013). Binary RDF representation for publication and exchange (HDT). Web Semant.: Sci. Serv. Agents World Wide Web.

[12] Fernández-Canellas, D., et al.: Linking Media: adopting Semantic Technologies for multimodal media connection (2018)

[13] Heitmann, B., Hayes, C.: Using linked data to build open, collaborative recommender systems. In: AAAI Spring Symposium: Linked Data Meets Artificial Intelligence (2010)

[14] Herrman, J.: Media websites battle faltering ad revenue and traffic. The New York Times (2016). Stated by Brian Nowak, a Morgan Stanley analyst

[15] Heyvaert P, De Meester B, Dimou A, Verborgh R, Gangemi A (2018). Declarative rules for linked data generation at your fingertips!. The Semantic Web: ESWC 2018 Satellite Events.

[16] Jabłonowska, A., Michałowicz, A.: Planet49: pre-ticked checkboxes are not sufficient to convey users consent to the storage of cookies (C-673/17 Planet49). Eur. Data Prot. Law Rev. **6**(1) (2020). 10.21552/edpl/2020/1/19

[17] Kirrane S, Gangemi A (2018). A scalable consent, transparency and compliance architecture. The Semantic Web: ESWC 2018 Satellite Events.

[18] Laperdrix, P., Rudametkin, W., Baudry, B.: Beauty and the beast: diverting modern web browsers to build unique browser fingerprints. In: 2016 IEEE Symposium on Security and Privacy (SP) (2016)

[19] Martínez-Prieto MA, Arias Gallego M, Fernández JD, Simperl E, Cimiano P, Polleres A, Corcho O, Presutti V (2012). Exchange and consumption of huge RDF data. The Semantic Web: Research and Applications.

[20] Miles, A., Bechhofer, S.: SKOS Simple Knowledge Organization System Reference. Recommendation, World Wide Web Consortium (W3C) (2009). https://www.w3.org/TR/skos-reference/

[21] Moulding, J.: Sky and Virgin prove the TV industry can set aside its rivalries to deliver the advanced ad-tech scale that buyers want. Videonet (2017)

[22] Nill A, Aalberts RJ (2014). Legal and ethical challenges of online behavioral targeting in advertising. J. Curr. Issues Res. Advert..

[23] Pandit HJ, Panetto H, Debruyne C, Hepp M, Lewis D, Ardagna CA, Meersman R (2019). Creating a vocabulary for data privacy. On the Move to Meaningful Internet Systems: OTM 2019 Conferences.

[24] van Someren MW, Barnard Y, Sandberg J (1994). The Think Aloud Method: A Practical Guide to Modelling Cognitive Processes. Knowledge-Based Systems.

[25] Sporny, M., Kellogg, G., Lanthaler, M.: JSON-LD 1.0 - a JSON-based serialization for linked data. Recommendation, World Wide Web Consortium (W3C) (2014). http://www.w3.org/TR/json-ld/

[26] Taelman R, Van Herwegen J, Vander Sande M, Verborgh R, Vrandečić D (2018). Comunica: a modular SPARQL query engine for the web. The Semantic Web – ISWC 2018.

[27] Thanapalasingam T, Osborne F, Birukou A, Motta E, Vrandečić D (2018). Ontology-based recommendation of editorial products. The Semantic Web – ISWC 2018.

[28] Thomas E, Pan JZ, Taylor S, Ren Y, Jekjantuk N, Zhao Y, Zseby T, Savola R, Pistore M (2010). Semantic advertising for web 3.0. Future Internet - FIS 2009.

[29] Verborgh R (2016). Triple Pattern Fragments: a low-cost knowledge graph interface for the Web. J. Web Semant..

[30] Wang, C., Zhang, P., Choi, R., D’Eredita, M.: Understanding consumers attitude toward advertising. In: 8th Americas Conference on Information Systems (2002)

[31] Zheng HT, Chen JY, Jiang Y (2012). An ontology-based approach to Chinese semantic advertising. Inf. Sci..

